# Mr. Syme’s Treatment of Callous Ulcers of the Extremities

**Published:** 1848-06

**Authors:** 


*Mr. Syme's Treatment of Callous Ulcers of the Extremities.*—Dissatisfied with the usual methods employed by surgeons, the author states that in 1829 he advised the application of "a large blister over the sore and neighbouring swelled parts of the limb, which has the effect of speedily dispersing the subcutaneous induration and thickening, so as to relax the integuments, and thus remove the obstacle to healing action. In the course of a short time, seldom exceeding a few days after the blister has been applied, the surface of the ulcer, however deep it may have been, is found to be on a level with that of the surrounding skin, not of course through any process of reproduction or filling up, but merely from the removal of interstitial effusion, allowing the integuments to descend from the position to which they had been elevated, as may be readily ascertained by measuring the circumference of the limb, before and after it has undergone the effect of blistering. But, along with this change of form, the ulcer in other respects no less speedily acquires the characters of a healing sore, assuming a florid colour, affording a moderate discharge of purulent matter, and presenting a granulating surface with surrounding margin of cicatrizing pellicle. No subsequent treatment beyond the attention requisite for ensuring quiet and cleanliness is needed, and recovery is completed, not only more quickly, but with much less tendency to relapse than when accomplished by other means.

"The facility, rapidity, economy, and lasting effect of this treatment, seem to give it a decided advantage over the other methods in use; and, so far as I am aware, no one who has tried the plan ever afterwards hesitated to employ it in preference to any other. In order to derive the full amount of benefit which the practice affords, it must be carried fairly into effect; and with this view, the principle upon which it is founded should be distinctly understood. I still entertain the opinion originally expressed, that the blisters act beneficially by inducing a process of absorption. The enlargement of the limb being of secondary formation, and resulting from the continued irritation of a sore allowed to remain unhealed through neglect or improper treatment, when once established, prevents the contraction of granulating action, by which alone solutions of continuity, not within reach of union by simple adhesion, admit of reparation. Pressure, the horizontal posture, and all other means that tend to remove the obstacle thus presented, will promote the patient's recovery. But of all the means that can be employed for this purpose, blisters appear to be the most efficient, and should therefore be employed for the remedy, not only of the purely indolent and callous ulcer, but of other kinds, which, in addition to their own peculiar characters, show evidence of complication with indurated enlargement of the limb. From this condition it is hardly necessary to mention that the œdematous swelling of weakness and impeded circulation must be distinguished."—*Contributions to the Pathology and Practice of Surgery.*

